# Research data management in academic institutions: A scoping review

**DOI:** 10.1371/journal.pone.0178261

**Published:** 2017-05-23

**Authors:** Laure Perrier, Erik Blondal, A. Patricia Ayala, Dylanne Dearborn, Tim Kenny, David Lightfoot, Roger Reka, Mindy Thuna, Leanne Trimble, Heather MacDonald

**Affiliations:** 1 Gerstein Science Information Centre, University of Toronto, Toronto, Ontario, Canada; 2 Institute of Health Policy, Management and Evaluation, University of Toronto, Toronto, Ontario, Canada; 3 Gibson D. Lewis Health Science Library, UNT Health Science Center, Fort Worth, Texas, United States of America; 4 St. Michael’s Hospital Library, St. Michael’s Hospital, Toronto, Ontario, Canada; 5 Faculty of Information, University of Toronto, Toronto, Ontario, Canada; 6 Engineering & Computer Science Library, University of Toronto, Toronto, Ontario, Canada; 7 Map and Data Library, University of Toronto, Toronto, Ontario, Canada; 8 MacOdrum Library, Carleton University, Ottawa, Ontario, Canada; International Nutrition Inc, UNITED STATES

## Abstract

**Objective:**

The purpose of this study is to describe the volume, topics, and methodological nature of the existing research literature on research data management in academic institutions.

**Materials and methods:**

We conducted a scoping review by searching forty literature databases encompassing a broad range of disciplines from inception to April 2016. We included all study types and data extracted on study design, discipline, data collection tools, and phase of the research data lifecycle.

**Results:**

We included 301 articles plus 10 companion reports after screening 13,002 titles and abstracts and 654 full-text articles. Most articles (85%) were published from 2010 onwards and conducted within the sciences (86%). More than three-quarters of the articles (78%) reported methods that included interviews, cross-sectional, or case studies. Most articles (68%) included the *Giving Access to Data* phase of the UK Data Archive Research Data Lifecycle that examines activities such as sharing data. When studies were grouped into five dominant groupings (Stakeholder, Data, Library, Tool/Device, and Publication), data quality emerged as an integral element.

**Conclusion:**

Most studies relied on self-reports (interviews, surveys) or accounts from an observer (case studies) and we found few studies that collected empirical evidence on activities amongst data producers, particularly those examining the impact of research data management interventions. As well, fewer studies examined research data management at the early phases of research projects. The quality of all research outputs needs attention, from the application of best practices in research data management studies, to data producers depositing data in repositories for long-term use.

## Introduction

Increased connectivity has accelerated progress in global research and estimates indicate scientific output is doubling approximately every ten years [1]. A rise in research activity results in an increase in research data output. However, data generated from research that is not prepared and stored for long-term access is at risk of being lost forever. Vines and colleagues report that the availability of data related to studies declines rapidly with the age of a study and determined that the odds of a data set being reported as available decreased 17% per year after publication)[2]. At the same time, research funding agencies and scholarly journals are progressively moving towards directives that require data management plans and demand data sharing [3–6]. The current research ecosystem is complex and highlights the need for focused attention on the stewardship of research data [1,7].

Academic institutions are multifaceted organizations that exist within the research ecosystem. Researchers practicing within universities and higher education institutions must comply with funding agency requirements when they are the recipients of research grants. For some disciplines, such as genomics and astronomy, persevering and sharing data is the norm [8–9] yet best practices stipulate that research be reproducible and transparent which indicates effective data management is pertinent to all disciplines.

Interest in research data management in the global community is on the rise. Recent activity has included the Bill & Melinda Gates Foundation moving their open access/open data policy, considered to be exceptionally strong, into force at the beginning of 2017 [10]. Researchers working towards a solution to the Zika virus organized themselves to publish all epidemiological and clinical data as soon as it was gathered and analyzed [11]. Fecher and colleagues [12] conducted a systematic review focusing on data sharing to support the development of a conceptual framework, however it lacked rigorous methods, such as the use of a comprehensive search strategy [13]. Another review on data sharing was conducted by Bull and colleagues [14] that examined stakeholders’ perspectives on ethical best practices but focused specifically on low- and middle-income settings. In this scoping review, we aim to assess the research literature that examines research data management as it relates to academic institutions. It is a time of increasing activity in the area of research data management [15] and higher learning institutions need to be ready to address this change, as well as provide support for their faculty and researchers. Identifying the current state of the literature so there is a clear understanding of the evidence in the area will provide guidance in planning strategies for services and support, as well as outlining essential areas for future research endeavors in research data management. The purpose of this study is to describe the volume, topics, and methodological nature of the existing research literature on research data management in academic institutions.

## Materials and methods

We conducted a scoping review using guidance from Arksey and O’Malley [16] and the Joanna Briggs Manual for Scoping Reviews [17]. A scoping review protocol was prepared and revised based on input from the research team, which included methodologists and librarians specializing in data management. It is available upon request from the corresponding author. Although traditionally applied to systematic reviews, the PRISMA Statement was used for reporting [18].

### Data sources and literature search

We searched 40 electronic literature databases from inception until April 3–4, 2016. Since research data management is relevant to all disciplines, we did not restrict our search to literature databases in the sciences. This was done in order to gain an understanding of the breadth of research available and provide context for the science research literature on the topic of research data management. The search was peer-reviewed by an experienced librarian (HM) using the Peer Review of Electronic Search Strategies checklist and modified as necessary [19]. The full literature search for MEDLINE is available in the S1 File. Additional database literature searches are available from the corresponding author. Searches were performed with no year or language restrictions. We also searched conference proceedings and gray literature. The gray literature discovery process involved identifying and searching the websites of relevant organizations (such as the Association of Research Libraries, the Joint Information Systems Committee, and the Data Curation Centre). Finally, we scanned the references of included studies to identify other potentially relevant articles. The results were imported into Covidence (covidence.org) for the review team to screen the records.

### Study selection

All study designs were considered, including qualitative and quantitative methods such as focus groups, interviews, cross-sectional studies, and randomized controlled trials. Eligible studies included academic institutions and reported on research data management involving areas such as infrastructure, services, and policy. We included studies from all disciplines within academic institutions with no restrictions on geographical location. Studies reporting results that accepted participants outside of academic institutions were included if 50% or more of the total sample represented respondents from academic institutions. For studies that examined entities other than human subjects, the study was included if the outcomes were pertinent to the broader research community, including academia. For example, if a sample of journal articles were retrieved to examine the data sharing statements but each study was not explicitly linked to a research sector, it was accepted into our review since the outcomes are significant to the entire research community and academia was not explicitly excluded. We excluded commentaries, editorials, or papers providing descriptions of processes that lacked a research component.

We define an academic institution as a higher education degree-granting organization dedicated to education and research. Research data management is defined as the storage, access, and preservation of data produced from a given investigation [20]. This includes issues such as creating data management plans, matters related to sharing data, delivery of services and tools, infrastructure considerations typically related to researchers, planners, librarians, and administrators.

A two-stage process was used to assess articles. Two investigators independently reviewed the retrieved titles and abstracts to identify those that met the inclusion criteria. The study selection process was pilot tested on a sample of records from the literature search. In the second stage, full-text articles of all records identified as relevant were retrieved and independently assessed by two investigators to determine if they met the inclusion criteria. Discrepancies were addressed by having a third reviewer resolve disagreements.

### Data abstraction and analysis

After a training exercise, two investigators independently read each article and extracted relevant data in duplicate. Extracted data included study design, study details (such as purpose, methodology), participant characteristics, discipline, and data collection tools used to gather information for the study. In addition, articles were aligned with the research data lifecycle proposed by the United Kingdom Data Archive [21]. Although represented in a simple diagram, this framework incorporates a comprehensive set of activities (creating data, processing data, analyzing data, preserving data, giving access to data, re-using data) and actions associated with research data management clarifying the longer lifespan that data has outside of the research project that is was created within (see S2 File). Differences in abstraction were resolved by a third reviewer. Companion reports were identified by matching the authors, timeframe for the study, and intervention. Those that were identified were used for supplementary material only. Risk of bias of individual studies was not assessed because our aim was to examine the extent, range, and nature of research activity, as is consistent with the proposed scoping review methodology [16–17].

We summarized the results descriptively with the use of validated guidelines for narrative synthesis [22–25]. Following guidance from Rodgers and colleagues, [22] data extraction tables were examined to determine the presence of dominant groups or clusters of characteristics by which the subsequent analysis could be organized. Two team members independently evaluated the abstracted data from the included articles in order to identify key characteristics and themes. Disagreement was resolved through discussion. Due to the heterogeneity of the data, articles and themes were summarized as frequencies and proportions.

## Results

### Literature search

**Fig 1:**
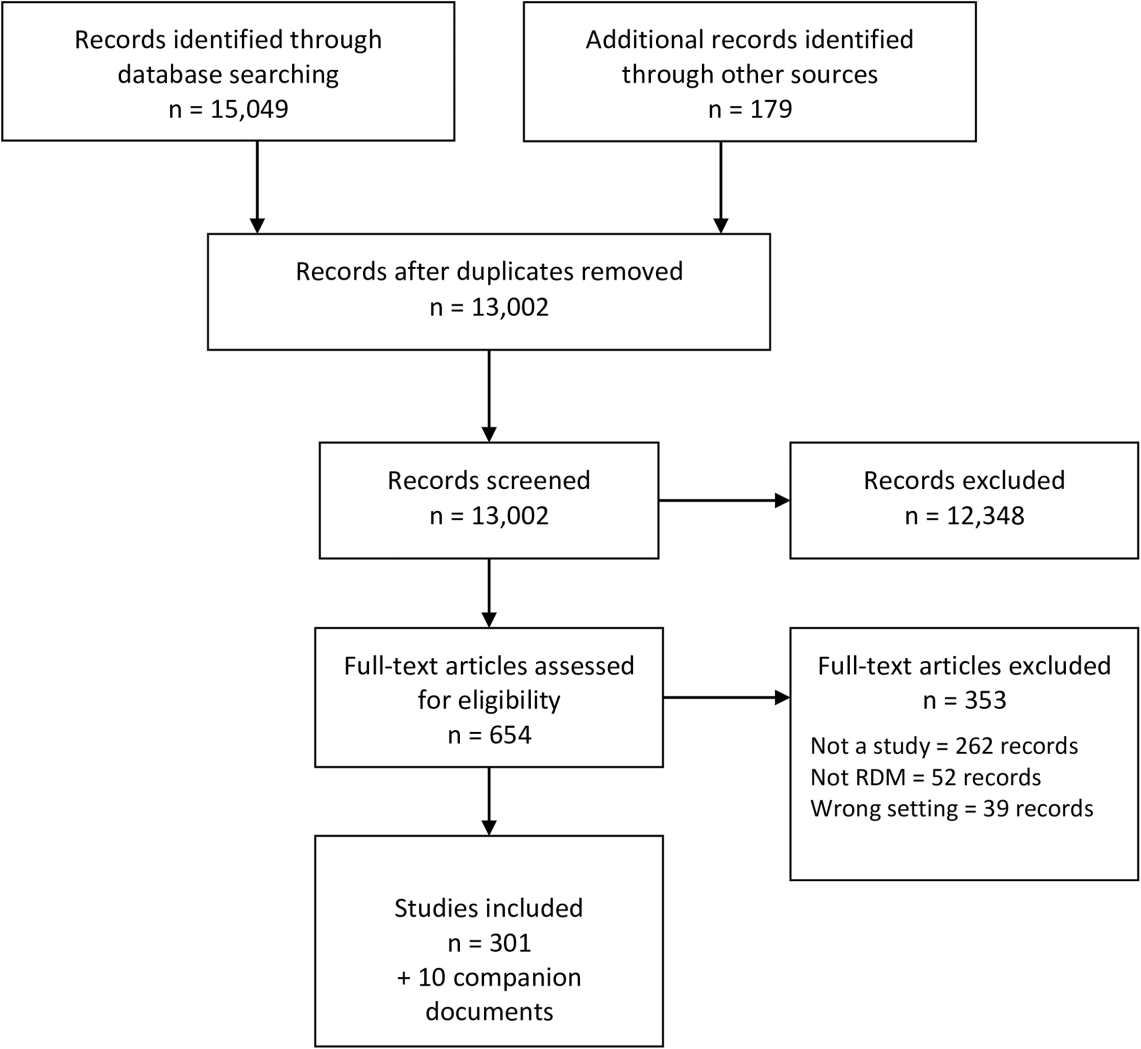
Flow diagram.

The literature search identified a total of 15,228 articles. After reviewing titles and abstracts, we retrieved 654 potentially relevant full-text articles. 301 articles were identified for inclusion in the study along with 10 companion documents (Fig 1). The full list of citations for the included studies can be found in the S3 File. The five literature databases that identified the most included studies were MEDLINE (81 articles or 21.60%), Compendex (60 articles or 16%), INSPEC (55 articles or 14.67%), Library and Information Science Abstracts (52 articles or 13.87%), and BIOSIS Previews (47 articles or 12.53%). The full list of electronic databases is available in the S4 File which also includes the number of included studies traced back to their original literature database.

### Characteristics of included articles

Most of the 301 articles were published from 2010 onwards (256 or 85.04%) with 15% published prior to that time (Table 1). Almost half (45.85%) identified North America (Canada, United States, or Mexico) as the region where studies were conducted; however, close to one fifth of articles (18.60%) did not report where the study was conducted. Most of the articles (78.51%) reported methods that included cross-sectional (129 or 35.54%), interviews (86 or 23.69%), or case studies (70 or 19.28%), with 42 articles (out of 301) describing two or more methods. Articles were almost even for reporting qualitative evidence (44.85%) and quantitative evidence (43.85%), with mixed methods representing a smaller proportion (11.29%). Reliance was put on authors in reporting characteristics of studies and no interpretations were made with regards to how attributes of the studies were reported. As a result, some information may appear to have overlap in the reporting of disciplines. For example, health science, medicine, and biomedicine are reported separately as disciplines/subject areas. Authors identified 35 distinct disciplines in the articles with just under ten percent (8.64%) not reporting a discipline and the largest group (105 or 34.88%) being a multidisciplinary. The two disciplines reported most often were medicine and information science/library science (31 or 10.30% each). Studies were reported in 116 journals, 43 conference papers, 26 gray literature documents (e.g., reports), two book chapters, and one PhD dissertation. Almost one-third of the articles (99 or 32.89%) did not use a data collection tool (e.g., when a case study was reported) and a small number (22 or 7.31%) based their data collection tools on instruments previously reported in the literature. Most data collection tools were either developed by authors (97 or 32.23%) or no description was provided about their development (83 or 27.57%). No validated data collection tools were reported. We identified articles that offered no information on the sample size or participant characteristics, [26–29] as well as those that reported on the number of participants that completed the study but failed to describe how many were recruited [30–31].

**Table 1:** Characteristics of included studies.

Characteristic	Articles, *n* (%); (*N* = 301) ^a^
**Year of publication**	
1995–2000	3 (1.00)
2001–2005	7 (2.33)
2006	8 (2.66)
2007	5 (1.66)
2008	9 (2.99)
2009	13 (4.32)
2010	24 (7.97)
2011	24 (7.97)
2012	46 (15.28)
2013	42 (13.95)
2014	58 (19.27)
2015	52 (17.28)
2016	10 (3.32)
**Geographic region**^b^	
North America	138 (45.85)
Europe	63 (20.93)
Other/not specified	56 (18.60)
Multi-continent	24 (7.97)
Australia	11 (3.65)
Asia	4 (1.33)
Africa	3 (1.00)
South America	2 (0.66)
**Study type**^c^	
Cross-sectional	129 (35.54)
Interviews	86 (23.69)
Case study	70 (19.28)
Content analysis	32 (8.82)
Focus groups	21 (5.79)
Bibliometric analysis	11 (3.30)
Ethnography	6 (1.65)
Usability study	2 (0.55)
Randomized controlled trial	2 (0.55)
Review (scoping or systematic)	2 (0.55)
Meta-analysis	1 (0.28)
**Type of evidence**	
Qualitative	135 (44.85)
Quantitative	132 (43.85)
Mixed methods (qualitative and quantitative)	34 (11.30)
**Discipline or subject area (as reported by authors of the articles)** ^**d**^	
Multidisciplinary	105 (34.88)
Medicine	31 (10.30)
Information science and library science	31 (10.30)
Other/not specified	26 (8.64)
Genetics	15 (4.98)
Ecology	11 (3.65)
Life sciences	8 (2.66)
Genomics	6 (1.99)
Health science	6 (1.99)
Social science	6 (1.99)
Biomedicine	5 (1.66)
Engineering	5 (1.66)
Science	5 (1.66)
Astronomy	4 (1.33)
Biology	4 (1.33)
Environmental science	4 (1.33)
Computer science	3 (1.00)
Agriculture	2 (0.66)
Archaeology	2 (0.66)
Chemistry	2 (0.66)
Earth science	2 (0.66)
Public health	2 (0.66)
Veterinary medicine	2 (0.66)
Agronomy	1 (0.33)
Animal behavior	1 (0.33)
Anthropology	1 (0.33)
Bioscience	1 (0.33)
Communication sciences	1 (0.33)
Crop science	1 (0.33)
Dance	1 (0.33)
Geography	1 (0.33)
Nanophotonics	1 (0.33)
Oceanography	1 (0.33)
Physics	1 (0.33)
Proteomics	1 (0.33)
Psychology	1 (0.33)
Sociology	1 (0.33)

^a^ Percentages may not total 100 because of rounding

^b^ Geographic region refers to where data originated, e.g., if telephone interviews were conducted with participants in France, Mexico, and Chile, the region would be listed as Multi-continent

^c^ Categories are not mutually exclusive, i.e., multiple study designs of two or more are reported in 42 articles

^d^ No attempt was made to create groupings, e.g., to collapse Chemistry and Science into one group

### Research data lifecycle framework

Two hundred and seven (31.13%) articles aligned with the *Giving Access to Data* phase of the Research Data Lifecycle [20] (Table 2) which include the components of distributing data, sharing data, controlling access, establishing copyright, and promoting data. The *Preserving Data* phase contained the next largest set of articles with 178 (26.77%). In contrast, *Analysing Data* and *Processing Data* were the two phases with the least amount of articles containing 28 (4.21%) and 49 (7.37%) respectively. Most articles (87 or 28.9%) were aligned with two phases of the Research Data Lifecycle and were followed by an almost even match of 73 (24.25%) aligning with three phases and 70 (23.26%) with one phase. Twenty-nine (9.63%) were not aligned with any phase of the Research Data Lifecycle and these included articles such as those that described education and training for librarians, or identified skill sets needed to work in research data management.

**Table 2:** Research data lifecycle.

Research Data Lifecycle Phase	Articles, *n* (%) ^a^
**Distribution of articles according to phase of Research Data Lifecycle** ^**b**^	
Creating Data *Components*: Design research, Plan data management, Plan consent for sharing (e.g., create consent forms), Locate existing data, Create data, Capture / create metadata	90 (13.53)
Processing Data *Components*: Enter data / digitize / transcribe / translate data, Check / validate / clean data, Anonymise data, Describe data, Manage / store data	49 (7.37)
Analysing Data *Components*: Interpret data, Derive data, Produce research outputs, Author publications, Prepare data for preservation	28 (4.21)
Preserving Data *Components*: Migrate data to best format, Migrate data to suitable medium, Back up / store data, Create metadata / documentation, Archive data	178 (26.77)
Giving Access to Data *Components*: Distribute data, Share data, Control access, Establish copyright, Promote data	207 (31.13)
Re-Using Data *Components*: Follow up research, New research, Undertake research reviews, Scrutinise findings, Teach and learn	113 (16.99)
**Number of phases represented per article (N = 301 articles)**	
1 phase	70 (23.26)
2 phase	87 (28.90)
3 phase	73 (24.25)
4 phase	20 (6.64)
5 phase	10 (3.32)
6 phase	12 (3.99)
No phases	29 (9.63)

Source: UK Data Archive, Research data lifecycle. Available at: http://data-archive.ac.uk/create-manage/life-cycle

^a^ Percentages may not total 100 because of rounding

^b^ Articles can be listed in more than one phase of the Research Data Lifecycle

### Key characteristics of articles

Five dominant groupings were identified for the 301 articles (Table 3). Each of these dominant groups were further categorized into subgroupings of articles to provide more granularity. The top three study types and the top three discipline/subject area is reported for each of the dominant groups. Half of the articles (151 or 50.17%) concentrated on stakeholders (Stakeholder Group), e.g., activities of researchers, publishers, participants / patients, funding agencies, 57 (18.94%) were data-focused (Data Group), e.g., investigating quality or integrity of data in repositories, development or refinement of metadata, 42 (13.95%) centered on library-related activities (Library Group), e.g., identifying skills or training for librarians working in data management, 27 (8.97%) described specific tools/applications/repositories (Tool/Device Group), e.g., introducing an electronic notebook into a laboratory, and 24 (7.97%) articles focused on the activities of publishing (Publication Group), e.g., examining data policies. The Stakeholder Group contained the largest subgroup of articles which was labelled ‘Researcher’ (119 or 39.53%).

**Table 3:** Groupings of articles.

Dominant Groups	Articles, *n* (%); (*N* = 301)^a^^,^^b^	Study Type (n) [top three listed]	Discipline or Subject Area (n) [top three listed]
**Stakeholder Group**	151 (50.17%)	1. Cross-sectional (survey) (76) 2. Interviews (58) 3. Case study (21)	1. Multidisciplinary (59) 2. Medicine (20) 3. Not reported (16)
*Subgroups*:			
* Researcher*	*119 (39*.*53)*		
* Institution / Administrator*	*17 (5*.*65)*		
* Participant / Patient*	*14 (4*.*65)*		
* Funder*	*1 (0*.*33)*		
**Data Group**	57 (18.94%)	1. Cross-sectional (survey) (14) 2. Interviews (13) 3. Case study (4)	1. Multidisciplinary (20) 2. Medicine (8) 3. Ecology (4) 3. Engineering (4)
*Subgroups*:			
* Data quality and integrity*	*21 (6*.*98)*		
* Repositories* *(includes characteristics*, *availability*, *awareness of)*	*11 (3*.*65)*		
* Classification systems* *(includes ontologies*, *refinement of metadata)*	*10 (3*.*32)*		
* Infrastructure and administration* *(includes Security/Privacy*, *Storage)*	*10 (3*.*32)*		
* Characteristics of specific disciplines*	*7 (2*.*33)*		
**Library Group**	42 (13.95%)	1. Case study (16) 2. Cross-sectional (survey) (14) 3. Interviews (10)	1. Information Science (26) 2. Multidisciplinary (8) 3. Medicine (2) 3. Not reported (2)
*Subgroups*:			
* Current status or assessment of needs* *(includes reporting on support or services offered)*	*28 (9*.*30)*		
* Skills required for librarians or data management personnel*	*9 (2*.*99)*		
* Training*	*8 (2*.*66)*		
**Tool/Device Group (specific tool, application, data repository)**	27 (8.97%)	1. Case study (22) 2. Cross-sectional (survey) (3) 3. Interviews (2) 3. Usability study (2) 3. Content analysis (2)	1. Multidisciplinary (7) 2. Not reported (5) 3. Information Science (2) 3. Environmental Science (2)
*Subgroups*:			
* Data management (tool*, *network)*	*16 (5*.*32)*		
* Data repository*	*13 (4*.*32)*		
**Publication Group**	24 (7.97%)	1. Cross-sectional (survey) (10) 2. Bibliometric study (7) 2. Content analysis (7) 3.Meta-analysis (1)	1. Multidisciplinary (7) 2. Genetics (5) 3. Genomics (3)
*Subgroups*:			
* Data policies*	*16 (5*.*32)*		
* Data availability*, *accessibility and reuse* *(includes author actual practice v*. *declared practice; data integrity)*	*16 (5*.*32)*		
* Citation rates*	*4 (1*.*33)*		

^a^ Percentages may not total 100 because of rounding

^b^ Articles can be listed in more than one grouping

## Discussion

We identified 301 articles and 10 companion documents that focus on research data management in academic institutions published between 1995 and 2016. Tracing articles back to their original literature database indicates that 86% of the studies accepted into our review were from the applied science or basic science literature indicating high activity for research in this area among the sciences. The number of published articles has risen dramatically since 2010 with 85% of articles published post-2009, signaling the increased importance and interest in this area of research. However, the limited use of study designs, deficiency in standardized or validated data collection tools, and lack of transparency in reporting demonstrate the need for attention to rigor. As well, there are limited studies that examine the impact of research data management activities (e.g., the implementation of services, training, or tools).

Few of the study designs employed in the 301 articles collected empirical evidence on activities amongst data producers such as examining changes in behavior (e.g., movement from data withholding to data sharing) or identifying changes in endeavors (e.g., strategies to increase data quality in repositories). Close to 80% of the articles rely on self-reports (e.g., participating in interviews, filling out surveys) or accounts from an observer (e.g., describing events in a case study). Case studies made up almost one-fifth of the articles examined. This group of articles ranged from question-and-answer journalistic style reports, [32] to articles that offered structured descriptions of activities and processes [33]. Although study quality was not formally assessed, this range of offerings provided challenges with data abstraction, in particular with the journalistic style accounts. If papers provided clear reporting that included declaring a purpose and describing well-defined outcomes, these articles could supply valuable input to knowledge syntheses such as a realist review [34–35] despite being ranked lower in the hierarchy of evidence [36]. One exception was Hruby and colleagues [37] that included a retrospective analysis in their case report that examined the impact of introducing a centralized research data repository for datasets within a urology department at Columbia University. This study offered readers a fuller understanding of the impact of a research data management intervention by providing evidence that detailed a change. Results described a reduction in the time required to complete studies, and an increase in publication quantity and quality (i.e., increase in average journal impact factor of papers published). There is opportunity for those wishing to conduct studies that provide empirical evidence for data producers and those interested in data reuse, however, for those wishing to conduct case studies, the development of reporting guidelines may be of benefit.

Using the Research Data Lifecycle framework provides the opportunity to understand where researchers are focusing their efforts in studying research data management. Most studies fell within the *Giving Access to Data* phase of the framework which includes activities such as sharing data and controlling access to data, and the *Preserving Data* phase which focuses on activities such as documenting and archiving data. This aligns with the global trend of funding agencies moving towards requirements for open access and open data [15] which includes activities such as creating metadata/documentation and sharing data in public repositories when possible. Fewer studies fell within phases that occurred at the beginning of the Research Data Lifecycle which includes activities such as writing data management plans and the preparation of data for preservation. Research in these early phases that include planning and setting up processes for handling data as it is being created may provide insight into how these activities impact later phases of the Research Data Lifecycle, in particular with regards to data quality.

Data quality was examined in several of the Groups described in Table 3. Within the Data Group, ‘data quality and integrity’ comprised the biggest subgroup of articles. Two other subgroups in the Data Group, ‘classification systems’ and ‘repositories’, provided articles that touched on issues related to data quality as well. These issues included refining metadata and improving functionalities in repositories that enabled scholarly use and reuse of materials. Willoughby and colleagues illustrated some of the challenges related to data quality when reporting on researchers in chemistry, biology, and physics [38]. They found that when filling out metadata for a repository, researchers used a ‘minimum required’ approach. The biggest inhibitor to adding useful metadata was the ‘blank canvas’ effect, where the users may have been willing to add metadata but did not know how. The authors concluded that simply providing a mechanism to add metadata was not sufficient. Data quality, or the lack thereof, was also identified in the Publication Group, with ‘data availability, accessibility, and reuse’ and ‘data policies’ subgroups listing articles that tracked the completeness of deposited data sets, and offered assessments on the guidance offered by journals on their data sharing policies. Piwowar and Chapman analyzed whether data sharing frequency was associated with funder and publisher requirements [39]. They found that NIH (National Institute of Health) funding had little impact on data sharing despite policies that required this. Data sharing was significantly association with the impact factor of a journal (not a journal’s data sharing policy) and the experience of the first/last authors. Studies that investigate processes to improve the quality of data deposited in repositories, or strategies to increase compliance with journal or funder data sharing policies that support depositing high-quality and useable data, could potentially provide tangible guidance to investigators interested in effective data reuse.

We found a number of articles with important information not reported. This included the geographic region in which the study was conducted (56 or 18.6%) and the discipline or subject area being examined (26 or 8.64%). Data abstraction identified studies that provided no information on participant populations (such as sample size or characteristics of the participants) as well as studies that reported the number of participants who completed the study, but failed to report the number recruited. Lack of transparency and poor documentation of research is highlighted in the recent Lancet series on ‘research waste’ that calls attention to avoiding the misuse of valuable resources and the inadequate emphasis on the reproducibility of research [40]. Those conducting research in data management must recognize the importance of research integrity being reflected in all research outputs that includes both publications and data.

## Conclusion

We identified a sizable body of literature that describes research data management related to academic institutions, with the majority of studies conducted in the applied or basic sciences. Our results should promote further research in several areas. One area includes shifting the focus of studies towards collecting empirical evidence that demonstrates the impact of interventions related to research data management. Another area that requires further attention is researching activities that demonstrate concrete improvements to the quality and usefulness of data in repositories for reuse, as well as the examining facilitators and barriers for researchers to participate in this activity. In particular, there is a gap in research that examines activities in the early phases of research projects to determine the impact of interventions at this stage. Finally, researchers investigating research data management must follow best practices in research reporting and ensure the high quality of their own research outputs that includes both publications and datasets.

## Supporting information

S1 File MEDLINE search strategy. (DOCX)

S2 File Research data lifecycle phases. (DOC)

S3 File Included studies. (DOCX)

S4 File Literature databases searched. (DOCX)
