# Supplementary material for: Research data management in academic institutions: A scoping review
Source: PLoS One. 2017 May 23;12(5):e0178261. doi: 10.1371/journal.pone.0178261 (PMC5441653; doi:10.1371/journal.pone.0178261)
Supplement: S1 File — (DOCX) [file pone.0178261.s001.docx]

## S1 File. MEDLINE Search Strategy.

Database: Ovid MEDLINE(R) <1946 to March Week 4 2016>, Ovid MEDLINE(R) In-Process & Other Non-Indexed Citations <April 01, 2016>

Search Strategy:

--------------------------------------------------------------------------------

1 Data Collection/ and Information Management/

2 Records as Topic/

3 Datasets as Topic/

4 "research data management".tw.

5 (accessib$ adj2 data$).tw.

6 (accessib$ adj2 research).tw.

7 (shar$ adj2 data$).tw.

8 (shar$ adj2 research).tw.

9 (transparen$ adj2 data).tw.

10 (transparen$ adj2 research).tw.

11 (cyber adj infrastructure).tw.

12 (reus$ adj2 data$).tw.

13 (reus$ adj2 research).tw.

14 (re-us$ adj2 data$).tw.

15 (re-us$ adj2 research).tw.

16 escience.tw.

17 e-science.tw.

18 (esocial adj science).tw.

19 (e-social adj science).tw.

20 eresearch.tw.

21 e-research.tw.

22 (research adj repository).tw.

23 (research adj repositories).tw.

24 (data$ adj repository).tw.

25 (data$ adj repositories).tw.

26 (data adj stewardship).tw.

27 (data adj curation).tw.

28 (data adj preservation).tw.

29 "open research data".tw.

30 cyberscholarship.tw.

31 cyber-scholarship.tw.

32 or/1-31

33 "Academies and Institutes"/

34 exp Libraries/

35 Library Services/

36 Universities/

37 exp Faculty/

38 exp Education, Graduate/

39 exp Academic Medical Centers/

40 academic.tw.

41 academia.tw.

42 library.tw.

43 libraries.tw.

44 university.tw.

45 universities.tw.

46 faculty.tw.

47 professor?.tw.

48 researcher.tw.

49 researchers.tw.

50 investigator?.tw.

51 scientist?.tw.

52 (graduate adj student?).tw.

53 (master? adj student?).tw.

54 (PhD adj student?).tw.

55 post-doc$.tw.

56 (research adj fellow$).tw.

57 or/33-56

58 32 and 57

59 exp Animals/ not Humans/

60 58 not 59
