# Supplementary material for: Research data management in academic institutions: A scoping review
Source: PLoS One. 2017 May 23;12(5):e0178261. doi: 10.1371/journal.pone.0178261 (PMC5441653; doi:10.1371/journal.pone.0178261)
Supplement: S2 File — (DOC) [file pone.0178261.s002.doc]

**S2 File. Research Data Lifecycle Phases.**

| 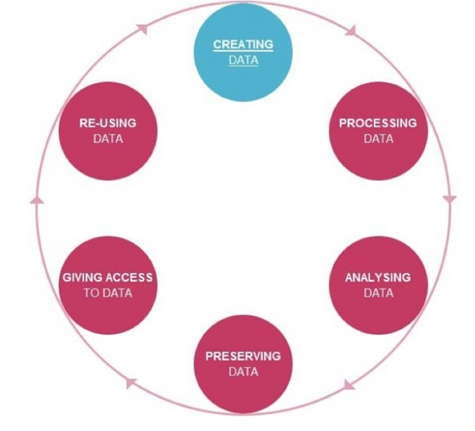 | | |
| --- | --- | --- |
| **Creating Data** | - Design research - Plan data management - Plan consent for sharing (e.g., create consent forms) | - Locate existing data - Create data - Capture / create metadata |
| **Processing Data** | - Enter data / digitize / transcribe / translate - Check / validate / clean data | - Anonymise data - Describe data - Manage / store data |
| **Analyzing Data** | - Interpret data - Derive data - Produce research outputs | - Author publications - Prepare data for preservation |
| **Preserving Data** | - Migrate data to best format - Migrate data to suitable medium - Back up / store | - Create metadata / documentation - Archive data |
| **Giving Access to Data** | - Distribute data - Share data - Control access | - Establish copyright - Promote data |
| **Re-Using Data** | - Follow up research - New research - Undertake research reviews | - Scrutinise findings - Teach / learn |

Source: UK Data Archive. Available at: http://www.data-archive.ac.uk/create-manage/life-cycle
