# Supplementary material for: Research data management in academic institutions: A scoping review
Source: PLoS One. 2017 May 23;12(5):e0178261. doi: 10.1371/journal.pone.0178261 (PMC5441653; doi:10.1371/journal.pone.0178261)
Supplement: S3 File — (DOCX) [file pone.0178261.s003.docx]

**S3 File. Included studies.**

**INCLUDED STUDIES**

1. Akers K, Doty J. Differences among faculty ranks in views on research data management. IASSIST Quarterly. 2012; 36(2):16.20.
2. Akers KG, Doty J. Disciplinary differences in faculty research data management practices and perspectives. International Journal of Digital Curation. 2013; 8(2):5.26.
3. Akers KG, Green JA. Towards a symbiotic relationship between academic libraries and disciplinary data repositories: a Dryad and University of Michigan case study. International Journal of Digital Curation. 2014; 9(1):119--131.
4. Akers KG, Sferdean FC, Nicholls NH, Green JA. Building Support for Research Data Management: Biographies of Eight Research Universities. International Journal of Digital Curation. 2014; 9(2):171-191.
5. Aleixandre-Benavent R, Vidal-Infer A, Alonso, Arroyo A, Valderrama ZJC, Bueno CF, Ferrer SA. Public availability of published research data in substance abuse journals. The International Journal on Drug Policy. 2014; 25(6):1143-1146.
6. Allard S, Aydinoglu AU. Environmental researchers' data practices: An exploratory study in Turkey. International Symposium on Information Management in a Changing World. Springer Berlin Heidelberg. 2012; 317:13-24.
7. Allen C, Foulkes WD. Qualitative thematic analysis of consent forms used in cancer genome sequencing. BMC Medical Ethics. 2011; 12:14.
8. Alsheikh-Ali AA, Qureshi W, Al-Mallah MH, Ioannidis JP. Public availability of published research data in high-impact journals. PLoS One. 2011; 6(9):e24357.
9. Alvaro E, Brooks H, Ham M, Poegel S, Rosencrans S. E-science librarianship: field undefined. Issues in Science and Technology Librarianship. 2011; no. 66.
10. Amorim RC, Castro JA, da Silva JR, Ribeiro C. A Comparative Study of Platforms for Research Data Management: Interoperability, Metadata Capabilities and Integration Potential. New Contributions in Information Systems and Technologies. Heidelberg: Springer International Publishing. 2015: 101-111.
11. Amorim RC, Castro JA, Dasilva JR, Ribeiro C. Engaging researchers in data management with labtablet, an electronic laboratory notebook. International Symposium on Languages, Applications and Technologie. 2015; 216-223.
12. Amos H, Frances M, Ruthven T. RSQUARED: researching the researchers: a study into how researchers at the University of New South Wales use and share research data. International Association of Scientific and Technological University Libraries, 31st Annual Conference. 2010.
13. Anagnostou P, Capocasa M, Milia N, Sanna E, Battaggia C, Luzi D, Destro Bisol G. When data sharing gets close to 100%: what human paleogenetics can teach the open science movement. PLoS One. 2015; 10(3):e0121409.
14. Anderson NR, Lee ES, Brockenbrough JS, Minie ME, Fuller S, Brinkley J, Tarczy-Hornoch P. Issues in biomedical research data management and analysis: needs and barriers. Journal of the American Medical Informatics Association. 2007; 14(4):478-488/
15. Antell K, Foote JB, Turner J, Shults B. Dealing with data: science librarians' participation in data management at association of research libraries institutions. College & Research Libraries. 2014; 75(4):557-574.
16. Arguillas F, Heslop J, Whyte A. Reviewing research data platform capabilities at Cornell Institute for Social and Economic Research (CISER). DCC RDM Services case studies. Edinburgh: Digital Curation Centre. 2015. Available at: http://www.dcc.ac.uk/resources/case-studies. Accessed January 6, 2017.
17. Averkamp S, Gu X. Report on the University Libraries’ Data Management Needs Survey. 2012. Available at: http://ir.uiowa.edu/cgi/viewcontent.cgi?article=1245&context=lib_pubs. Accessed January 6, 2017.
18. Aydinoglu AU, Suomela T, Malone J. Data management in astrobiology: challenges and opportunities for an interdisciplinary community. Astrobiology. 2014; 14(6):451-461.
19. Bamkin M. Report of Findings from Focus Group and Online Questionnaire: The opinions of potential users of a policy databank service.
    Report of Findings from Focus Group and Online Questionnaire: The opinions of potential users of a policy databank service
    Report of Findings from Focus Group and Online Questionnaire: The opinions of potential users of a policy databank service. JORD Project. 2014. Available at: https://jordproject.files.wordpress.com/2014/06/report-of-findings-from-focus-group-and-online-questionnaire.pdf. Accessed January 6, 2017.
20. Bardyn TP, Resnick T, Camina SK. Translational researchers' perceptions of data management practices and data curation needs: findings from a focus group in an academic health sciences library. Journal of Web Librarianship. 2012; 6(4):274-287.
21. Belter CW. Measuring the value of research data: a citation analysis of oceanographic data sets. PLoS One. 2014; 9(3):e92590.
22. Berlinicke CA, Ackermann CF, Chen SH, Schulze C, Shafranovich Y, Myneni S, Patel VL, Wang J, Zack DJ, Lindvall M, Bova GS. High-content screening data management for drug discovery in a small- to medium-size laboratory: results of a collaborative pilot study focused on user expectations as indicators of effectiveness. Journal of Laboratory Automation. 2012; 14(4):255-265.
23. Beskow LM, Dean E. Informed Consent for Biorepositories: Assessing Prospective.
    Participants’ Understanding and Opinions. Cancer Epidemiology, Biomarkers & Prevention. 2008; 17(6):1440-1451.
24. Beskow LM, Friedman JY, Hardy NC, Lin L, Weinfurt KP. Simplifying informed consent for biorepositories: stakeholder perspectives. Genetics In Medicine. 2010; 12(9):567-572.
25. Bigagli L, Sveinsdottir T, Wessels B, Smallwood R, Linde P, Sondervan J. Policy RECommendations for Open access to research Data in Europe. 2014. Avaiable at: www.recodeproject.eu. Accessed January 6, 2017.
26. Bishoff C, Johnston L. Approaches to data sharing: an analysis of NSF data management plans from a large research university. Journal of Librarianship & Scholarly Communication. 2015; 3(2):e1231.
27. Blumenthal D, Campbell EG, Anderson MS, Causino N, Louis KS. Withholding research results in academic life science. Evidence from a national survey of faculty. JAMA. 1997; 277(15):1224-1228.
28. Blumenthal D, Campbell EG, Causino N, Louis KS. Participation of life-science faculty in research relationships with industry. New England Journal of Medicine. 1996; 335(23):1734-9.
29. Blumenthal D, Campbell EG, Gokhale M, Yucel R, Clarridge B, Hilgartner S, Holtzman NA. Data withholding in genetics and the other life sciences: prevalences and predictors. Academic Medicine. 2006; 81(2):137-145.
30. Blumenthal D, Causino N, Campbell EG. Academic-industry research relationships in genetics: a field apart. Nature Genetics. 1997; 16(1):104-108.
31. Bohemier KA, Atwood T, Kuehn A, Qin J. A content analysis of institutional data policies. Proceedings of the 11th Annual International ACM/IEEE Joint Conference on Digital Libraries. 2011; 409-410.
32. Borgman CL, Darch PT, Sands AE, Pasquetto IV, Golshan MS, Wallis JC, Traweek S. Knowledge infrastructures in science: data, diversity, and digital libraries. International Journal on Digital Libraries. 2015; 16:207-227.
33. Borgman CL, Wallis JC, Mayernik MS. Who's got the data? Interdependencies in science and technology collaborations. Computer Supported Cooperative Work: CSCW: An International Journal. 2012; 21(6):485-523.
34. Bracke MS. Emerging data curation roles for librarians: a case study of agricultural data. Journal of Agricultural & Food Information. 2011; 12(1):65-74.
35. Bradbury S, Borchert M. Survey of eResearch practices and skills at QUT, Australia. Proceedings of 31st Annual IATUL Conference: The Evolving World of e-Science: Impact and Implications for Science and Technology Libraries. 2010.
36. Bradic-Martinovic A, Zdravkovic A. Researchers’ Interest in Data Service in Bosnia and Herzegovina, Croatia, and Serbia. IASSIST Quarterly. 2014; 23.
37. Brandt SD, Kim E. Data curation profiles as a means to explore managing, sharing, disseminating or preserving digital outcomes. International Journal of Performance Arts and Digital Media. 2014; 10(1):21-34.
38. Bresnahan MM, Johnson AM. Assessing scholarly communication and research data training needs. Reference Services Review. 2013; 41(3):413-433.
39. Brewerton G. Research data management: A case study. Ariadne. 2015; 74. Available at: http://www.ariadne.ac.uk/issue74/brewerton. Accessed January 6, 2017.
40. Briney K, Goben A, Zilinksi L. Do You Have an Institutional Data Policy? A Review of the Current Landscape of Library Data Services and Institutional Data Policies. Journal of Librarianship and Scholarly Communication. 2015; 3(2):e1232.
41. Brooking C, Shouldice SR, Robin G, Kobe B, Martin JL, Hunter J. Comparing METS and OAI-ORE for encapsulating scientific data products: a protein crystallography case study. Fifth IEEE International Conference. 2009; 148-55.
42. Broom A, Cheshire L, Emmison M. Qualitative researchers' understandings of their practice and the implications for data archiving and sharing. Sociology. 2009; 43(6):1163-1180.
43. Bruin RP, Dove MT, Calleja M, Tucker MG. Building and managing the eminerals clusters: A case study in grid-enabled cluster operation. Computing in Science and Engineering. 2005; 7(6):30-37.
44. Budroni P, Solis BS. Factors for Enabling Sharing and Reuse of Research Data – Library and Archive Services at the University of Vienna. LIBER Quarterly. 2014. Available at: http://libereurope.eu/wp-content/uploads/2014/06/LIBER-Case-Study-UNVIE1.pdf. Accessed January 6, 2017.
45. Bull S, Roberts N, Parker M. Views of Ethical Best Practices in Sharing Individual-Level Data From Medical and Public Health Research: A Systematic Scoping Review. Journal of Empirical Research on Human Research Ethics. 2015; 10(3):225-38.
46. Burge S, Attwood TK, Bateman A, Berardini TZ, Cherry M, O'Donovan C, Xenarios L, Gaudet P. Biocurators and biocuration: surveying the 21st century challenges. Database. 2012; 2012:bar059.
47. Buys C, Shaw P. Data Management Practices Across an Institution: Survey and Report. Journal of Librarianship and Scholarly Communication. 2015; 3(2):e1225.
48. Caetano D, Aisenberg A. Forgotten treasures: the fate of data in animal behaviour studies. Animal Behaviour. 2014; 98:1-5.
49. Campbell EG, Bendavid E. Data-sharing and data-withholding in genetics and the life sciences: results of a national survey of technology transfer officers. Journal of Health Care Law & Policy. 2003; 6(2):241-255.
50. Campbell HA, Beyer HL, Dennis TE, Dwyer RG, Forester JD, Fukuda Y, Lynch C, Hindell MA, Menke N, Morales JM, Richardson C, Rodgers E, Taylor G, Watts ME, Westcott DA. Finding our way: on the sharing and reuse of animal telemetry data in Australasia. Science of the Total Environment. 2015; 534:79-84.
51. Campbell, EG, Clarridge BR, Gokhale M, Birenbaum L, Hilgartner S, Holtzman NA, Blumenthal D. Data withholding in academic genetics: evidence from a national survey. JAMA. 2002; 287(4):473-480.
52. Capocasa M, Anagnostou P, D'Abramo F, Matteucci G, Dominici V, Destro Bisol G, Rufo F. Samples and data accessibility in research biobanks: an explorative survey. PeerJ. 2016; 4:e1613.
53. Carlson J, Fosmire M, Miller CC, Nelson MS. Determining data information literacy needs: a study of students and research faculty. portal: Libraries and the Academy. 2011; 11(2):629-657.
54. Carlson J, Stowell-Bracke M. Data management and sharing from the perspective of graduate students: an examination of the culture and practice at the water quality field station. portal: Libraries and the Academy. 2013; 13(4):343-361.
55. Castro JA, Perrotta D, Amorim RC, da Silva JR, Ribeiro C. Ontologies for Research Data Description: A Design Process Applied to Vehicle Simulation.
     Metadata and Semantics Research. 2015; 348-354.
56. Castro JA, Ribeiro C, Da Silva, JR. Designing an application profile using qualified Dublin core: A case study with fracture mechanics datasets. Proceedings of the DC-2013 Conference. 2013; 47-52.
57. Chad K, Enright S. The research cycle and research data management (RDM): innovating approaches at the University of Westminster. Insights. 2014; 27(2):147-153.
58. Chao TC. Exploring the rhythms of scientific data use. Proceedings of the 2012 iConference. 2012; 129-135.
59. Charbonneau DH, Beaudoin JE. The State of Data Guidance in Journal Policies: A CaseStudy in Oncology. International Journal of Digital Curation. 2015; 10(2):136-156.
60. Cheah PY, Tangseefa D, Somsaman A, Chunsuttiwat T, Nosten F, Day NPJ, Bull S, Parker M. Perceived benefits, harms, and views about how to share data responsibly: a qualitative study of experiences with and attitudes toward data sharing among research staff and community representatives in Thailand. Journal of Empirical Research on Human Research Ethics. 2015; 10(3):278-289.
61. Cheikhi L, Abran A. Promise and ISBSG software engineering data repositories: a survey. Software Measurement and the 2013 Eighth International Conference on Software Process and Product Measurement (IWSM-MENSURA), 2013 Joint Conference of the 23rd International Workshop. 2013; 17-24.
62. Chen, HH, Lin Y, Chen C. Approaches to building metadata for data curation. Proceedings of the International Conference on Dublin Core and Metadata Applications. 2013; 190-193.
63. Choudhury GS. Case study in data curation at Johns Hopkins University. Library Trends. 2008; 57(2):211-220.
64. Colledge F, Persson K, Elger B, Shaw D. Sample and data sharing barriers in biobanking: consent, committees, and compromises. Annals of Diagnostic Pathology. 2014; 18(2):78-81.
65. Collins E. Publishing priorities of biomedical research funders. BMJ Open. 2013; 3(10):e004171.
66. Conklin JL. Identifying liaison opportunities through content analysis: academic library trends in the Ecological Society of America's conference program. Issues in Science & Technology Librarianship. 2013; (73):7.
67. Conner LG, Ames DP, Gill RA. HydroServer Lite as an open source solution for archiving and sharing environmental data for independent university labs. Ecological Informatics. 2013; 18:171-177.
68. Cooper M. Sharing data and results with study participants: report on a survey of cultural anthropologists. Journal of Empirical Research on Human Research Ethics. 2008; 3(4):19-34.
69. Corrall S, Kennan MA, Afzal W. Bibliometrics and research data management services: emerging trends in library support for research. Library Trends. 2013; 61(3):636-674.
70. Cox A, Verbaan E, Sen B. Upskilling liaison librarians for research data management. Ariadne. 2012; 70. Available at: http://www.ariadne.ac.uk/issue70/cox-et-al.
71. Cox AM, Pinfield S, Smith J. Moving a brick building: UK libraries coping with research data management as a ‘wicked’ problem. Journal of Librarianship and Information Science. 2016; 48(1):3-17.
72. Cox AM, Pinfield S. Research data management and libraries: current activities and future priorities. Journal of Librarianship and Information Science. 2014; 46(4):299-316.
73. Cragin MH, Palmer CL, Carlson JR, Witt M. Data sharing, small science and institutional repositories. Philosophical transactions. Series A, Mathematical, physical, and engineering sciences. 2010; 368(1926):4023-4038.
74. Creamer A, Morales M, Crespo J, Kafel D, Martin ER. Assessment of health sciences and science and technology librarian e-science educational needs to develop an e-science web portal for librarians. Journal of the Medical Library Association. 2011; 99(2):153-156.
75. Cugler DC, Medeiros CB, Shekhar S, Toledo LF. A geographical approach for metadata quality improvement in biological observation databases. IEEE 9th International Conference. 2013; 212-220.
76. Daniels M, Faniel I, Fear K, Yakel E. Managing fixity and fluidity in data repositories. Proceedings of the 2012 iConference. 2012; 279-286.
77. Darch PT, Borgman CL, Traweek S, Cummings RL, Wallis JC, Sands AE. What lies beneath?: knowledge infrastructures in the subseafloor biosphere and beyond. International Journal on Digital Libraries. 2015; 16(1):61-77.
78. Dearborn CC, Barton AJ, Harmeyer NA. The Purdue University research repository: HUBzero customization for dataset publication and digital preservation. OCLC Systems & Services: International Digital Library Perspectives. 2014; 30(1):15-27.
79. Delasalle J. Research data management at the University of Warwick: recent steps towards a joined-up approach at a UK university. LIBREAS. Library Ideas. 2013; 23. Available at: http://libreas.eu/ausgabe23/10delasalle. Accessed January 6, 2017.
80. den Besten M, Thomas AJ, Schroeder R. Life science research and drug discovery at the turn of the 21st century: the experience of SwissBioGrid. Journal of Biomedical Discovery and Collaboration. 2009; 4:5-20.
81. Denison T, Stillman L. Academic and ethical challenges in participatory models of community research. Information, Communication & Society. 2012; 15(7):1037-1054.
82. Denny SG, Silaigwana B, Wassenaar D, Bull S, Parker M. Developing ethical practices for public health research data sharing in South Africa: the views and experiences from a diverse sample of research stakeholders. Journal of Empirical Research on Human Research Ethics. 2015; 10(3):290-301.
83. Diekema AR, Wesolek A, Walters CD. The NSF/NIH effect: surveying the effect of data management requirements on faculty, sponsored programs, and institutional repositories The Journal of Academic Librarianship. 2014; 40:322-331.
84. Diekmann F. Data practices of agricultural scientists: results from an exploratory study. Journal of Agricultural & Food Information. 2012; 13(1):14-34.
85. Dietrich D, Adamus T, Miner A, Steinhart G. De-mystifying the data management requirements of research funders. Issues in Science and Technology Librarianship. 2012; 70. Available online: http://www.istl.org/12-summer/refereed1.html.
86. Drew BT, Gazis R, Cabezas P, Swithers KS, Deng J, Rodriguez R, Katz LA, Crandall KA, Hibbett DS, Soltis DE. Lost branches on the tree of life. PLoS Biology. 2013; 11(9):e1001636.
87. Ducloy J, Ducasse JP, Foulonneau Muriel, Grivel L, Le Henaff D, Nicolas Y. Metadata towards an e-research cyberinfrastructure the case of French PhD theses. Proceedings of the 2006 International Conference on Dublin Core and Metadata Applications. 2006; 1-10.
88. Edwards KL, Lemke AA, Trinidad SB, Lewis SM, Starks H, Quinn Griffin MT, Wiesner GL. Attitudes toward genetic research review: results from a survey of human genetics researchers. Public Health Genomics. 2011; 14(6):337-345.
89. Einbinder JS, Scully KW, Pates RD, Schubart JR, Reynolds RE. Case study: a data warehouse for an academic medical center. Journal of Healthcare Information Management. 2001; 15(2):165-175.
90. Embi PJ, Hebert C, Gordillo G, Kelleher K, Payne PRO. Knowledge management and informatics considerations for comparative effectiveness research: a case-driven exploration. Medical Care. 2013; 51(8):S38-44.
91. Enke N, Thessen A, Bach K, Bendix J, Seeger B, Gemeinholzer B. The user's view on biodiversity data sharing - investigating facts of acceptance and requirements to realize a sustainable use of research data. Ecological Informatics. 2012; 11:25-33.
92. Eschenfelder KR, Johnson A. Managing the data commons: controlled sharing of scholarly data. Journal of the Association for Information Science and Technology. 2014; 65(9):1757-1774.
93. Faniel I, Kansa E, Kansa SW, Barrera-Gomez J, Yakel E. The challenges of digging data: A study of context in archaeological data reuse. Proceedings of the 13th ACM/IEEE-CS Joint Conference on Digital Libraries. 2013; 295-304.
94. Faniel IM, Jacobsen TE. Reusing scientific data: how earthquake engineering researchers assess the reusability of colleagues data. Computer Supported Cooperative Work. 2010; 19:355-375.
95. Fear K, Donaldson DR. Provenance and credibility in scientific data repositories. Archival Science. 2012; 12(3):319-339.
96. Fecher B, Friesike S, Hebing M. What drives academic data sharing? PLoS One. 2015; 10(2):e0118053.
97. Federer L. The librarian as research informationist: a case study. Journal of the Medical Library Association. 2013; 101(4):298.
98. Fielding NG. Grid Computing and Qualitative Social Science. Social Science Computer Review. 2008; 26(3):301-16.
99. Finn R, Wadhwa K, Taylor M, Sveinsdottir T, Noorman M, Sondervan J. Legal and ethical issues in open access and data dissemination and preservation. Recode Project. 2014. Available at: www.recodeproject.eu. Accessed January 6, 2017.
100. Flechais I, Sasse MA. Stakeholder involvement, motivation, responsibility, communication: how to design usable security in e-science. International Journal of Human-Computer Studies. 2009; 67(4):281-96.
101. Frank RD, Yakel E, Faniel IM. Destruction/reconstruction: preservation of archaeological and zoological research data. Archival Science. 2015; 15(2):141-167.
102. Fry J, Schroeder R, den Besten M. Open science in e-science: contingency or policy? Journal of Documentation. 2009; 65(1):Jun-32.
103. Garritano JR, Carlson JR. A subject librarian's guide to collaborating on e-science projects. Issues in Science and Technology Librarianship. 2009; (57):16.
104. Goldenberg AJ, Maschke KJ, Joffe S, Botkin JR, Rothwell E, Murray TH, Anderson R, Deming N, Rosenthal BF, Rivera SM. IRB practices and policies regarding the secondary research use of biospecimens. BMC Medical Ethics. 2015; 16:32.
105. Goldman J, Kafel D, Marin ER. Assessment of Data Management Services at New England Region Resource Libraries. Journal of eScience Librarianship. 2015; 4(1):e1068.
106. Grace S, Whyte A, Rans J. Using EPrints to Build a Research Data.
     Repository for UEL DCC RDM Services case studies. Edinburgh: Digital Curation Centre. 2015; DCC RDM Services case studies. Edinburgh: Digital Curation Centre. Available at: http://www.dcc.ac.uk/resources/case-studies. Accessed January 6, 2017.
107. Green HE, Courtney A. Beyond the scanned image: a needs assessment of scholarly users of digital collections. College & Research Libraries. 2015; 76(5):690-707.
108. Grubb AM, Easterbrook SM. On the lack of consensus over the meaning of openness: an empirical study. PLoS One. 2011; 6(8):e23420.
109. Guy M. RDM Training for Librarians. DCC RDM Services case studies. Edinburgh: Digital Curation Centre. 2013; DCC RDM Services case studies. Edinburgh: Digital Curation Centre. Available at: http://www.dcc.ac.uk/resources/developing-rdm-services. Accessed January 6, 2017.
110. Haendel MA, Vasilevsky NA, Wirz JA. Dealing with Data: A Case Study on Information and Data Management Literacy. PLoS Biology. 2012; 10(5):e1001339.
111. Hall N. Environmental studies faculty attitudes towards sharing of research data. Proceedings of the 13th ACM/IEEE-CS Joint Conference on Digital Libraries. 2013; 383-384.
112. Hanauer DA, Hruby GW, Fort DG, Rasmussen LV, Mendonca EA, Weng C. What is asked in clinical data request forms? A multi-site thematic analysis of forms towards better data access support. AMIA Annu Symp Proc. 2014; 2014:616-625
113. Harris-Pierce RL, Quan Liu Y. Is data curation education at library and information science schools in North America adequate? New Library World. 2012; 113:598-613.
114. Hens K, Nys H, Cassiman JJ, Dierickx K. The use of diagnostic collections of DNA for research: interviews at the eight Belgian centers for human genetics. European Journal of Medical Genetics. 2010; 53(5):274-279.
115. Henty M, Weaver B, Bradbury SJ, Porter S. Investigating Data Management Practices in Australian Universities. 2008. Available at: http://eprints.qut.edu.au/14549. Accessed January 6, 2017.
116. Hernandez R, Mayernik M, Murphy-Mariscal M; Allen M. Advanced technologies and data management practices in environmental science: lessons from academia. BioScience. 2012; 62(12):1067-1076.
117. Herold P. Data Sharing Among Ecology, Evolution, and Natural Resources Scientists: An Analysis of Selected Publications. Journal of Librarianship and Scholarly Communication. 2015; 3(2):1-23.
118. Higman R, Pinfield S. Research data management and openness: the role of data sharing in developing institutional policies and practices. Program: Electronic Library and Information Systems. 2015; 49(4):364-381.
119. Hinnant CC, Stvilia B, Wu S, Worrall A, Burnett K, Burnett G, Kazmer MM, Marty PF. Data curation in scientific teams: An exploratory study of condensed matter physics at a national science lab. Proceedings of the 2012 iConference. 2012; 498-500.
120. Hiom D, Fripp D, Gray S, Snow K, Steer D. Research data management at the University of Bristol. Program. 2015; 49(4):475-493.
121. Hou CY, Thompson CA, Palmer CL. Profiling open digital repositories in the atmospheric and climate sciences: an initial survey. Proceedings of the Association for Informaiton Science and Technology. 2014; 51(1):1-4.
122. Hruby, GW, McKiernan J, Bakken S, Weng C. A centralized research data repository enhances retrospective outcomes research capacity: a case report. Journal of the American Medical Informatics Association. 2013; 20(3):563-567.
123. Huang H, Jorgensen C, Stivilia B. Geneomics data curation roles, skills and perception of data quality. Library & Information Science Research. 2015; 37(1):10-20.
124. Huang H, Stvilia B, Jorgensen C, Bass HW. Prioritization of data quality dimensions and skills requirements in genome annotation work. Journal of the American Society for Information Science and Technology. 2012; 63(1):195.
125. Huang X, Hawkins BA, Lei F, Miller GL, Favret C, Zhang R, Qiao G. Willing or unwilling to share primary biodiversity data: results and implications of an international survey. Conservation Letters. 2012; 5(5):399-406.
126. Ioannidis JP, Allison DB, Ball CA, Coulibaly I, Cui X, Culhane AC, Falchi M, Furlanello C, Game L, Jurman G, Mangion J, Mehta T, Nitzberg M, Page GP, Petretto E, van Noort V. Repeatability of published microarray gene expression analyses. Nature Genetics. 2009; 41(2):149-55.
127. Ishida M. The New England Collaborative Data Management Curriculum Pilot at the University of Manitoba: A Canadian Experience. Journal of eScience Librarianship. 2014; 3(1):10.
128. Jetten M. Research Data Management at Radboud University. LIBER Case Study. 2014. Available at: http://libereurope.eu/wp-content/uploads/2014/06/LIBER-Case-Study-Radboud.pdf. Accessed January 6, 2017.
129. Johnston L, Jeffryes J. Data management skills needed by structural engineering students: case study at the University of Minnesota. Journal of Professional Issues in Engineering Education and Practice. 2014; 140(2):05013002.
130. Johnston L. User-needs assessment of the research cyberinfrastructure for the 21st century. International Association of Scientific and Technological University Libraries, 31st Annual Conference. 2010; Paper 5.
131. Jones S. Bringing it all together: a case study on the improvement of Research Data Management at Monash University. DCC RDM Services case studies. Edinburgh: Digital Curation Centre. 2013. Available at: http://www.dcc.ac.uk/resources/developing-rdm-services. Accessed January 6, 2017.
132. Kansa EC, Kansa SW, Arbuckle B. Publishing and Pushing: Mixing Models forCommunicating Research Data in Archaeology. International Journal of Digital Curation. 2014; 9(1):57-70.
133. Karasti H. Baker KS, Halkola E. Enriching the notion of data curation in e-science: data managing and information infrastructuring in the long term ecological research (LTER) network. Computer Supported Cooperative Work: The Journal of Collaborative Computing. 2006; 15(4):321-358.
134. Kennan MA, Corrall S, Afzal W. "Making space" in practice and education: research support services in academic libraries. Library Management. 2014; 35(8/9):666
135. Kennan MA, Williamson K, Johanson G. Wild data: collaborative e-research and university libraries. Australian Academic & Research Libraries. 2012; 43(1):56-79.
136. Kerby EE. Research Data Practices in Veterinary Medicine: A Case Study. Journal of eScience Librarianship. 2015; 4(1):e1073.
137. Kervin K, Finholt T, Hedstrom M. Macro and micro pressures in data sharing. IEEE 13th International Conference. 2012; 525-32.
138. Kervin KE, Michener WK, Cook RB. Common Errors in Ecological Data Sharing. Journal of eScience Librarianship. 2013; 2(2):1.
139. Killeen NEB, Lohrey JM, Farrell M, Liu W, Garic S, Abramson D, Hoang N, Egan G. Integration of modern data management practice with scientific workflows. IEEE 8th International Conference on E-Science. 2012; 1-8.
140. Kim Y, Addom BK, Stanton JM. Education for eScience Professionals: Integrating Data Curation and Cyberinfrastructure. International Journal of Digital Curation. 2011; 6(1):125-138.
141. Kim Y, Adler M. Social scientists' data sharing behaviors: investigating the roles of individual motivations, institutional pressures, and data repositories. International Journal of Information Management. 2015; 35(4):408.
142. Kim Y, Burns CS. Norms of data sharing in biological sciences: the roles of metadata, data repository, and journal and funding requirements. Journal of Information Science. 2016; 42(2):230.
143. Kim Y, Stanton JM. Institutional and individual factors affecting scientists' data-sharing behaviors: a multilevel analysis. Journal of the Association for Information Science and Technology. 2016; 67(4):776.
144. Kim Y, Stanton JM. Institutional and Individual Influences on Scientists’ Data Sharing Practices. Journal of Computational Science Education. 2012; 3(1):47-56.
145. Kirlew PW. Life science data repositories in the publications of scientists and librarians. Issues in Science & Technology Librarianship. 2011; 65:5.
146. Knight G. A Digital Curate’s Egg: A Risk Management Approach to Enhancing Data Management Practices. Journal of Web Librarianship. 2012; 6(4):225-250.
147. Knight G. Building a research data management service for the London School of Hygiene & Tropical Medicine. Program: electronic library and information systems. 2015; 49(4):424-439.
148. Kowalczyk ST. Towards a model of the e-science data environment. 11th Annual International ACM/IEEE Joint Conference on Digital Libraries. 2011; 399-400.
149. Kratz JE, Strasser C. Researcher perspectives on publication and peer review of data. PLoS One. 2015; 10(2):e0117619.
150. Kruse F, Thestrup JB. Research libraries’ new role in research data management, current trends and visions in Denmark. LibER Quarterly. 2014; 23(4):310-335.
151. Kuchinke W, Ohmann C, Yang Q, Salas N, Lauritsen J, Gueyffier F, Leizorovicz A, Schade-Brittinger C, Wittenberg M, Voko Z, Gaynor S, Cooney M, Doran P, Maggioni A, Lorimer A, Torres F, McPherson G, Charwill J, Hellstrom M, Lejeune S. Heterogeneity prevails: the state of clinical trial data management in Europe - results of a survey of ECRIN centres. Trials. 2010; 11:79
152. Kutay S. Advancing digital repository services for faculty primary research assets: an exploratory study. The Journal of Academic Librarianship. 2014; 40(6):642-649.
153. Lage K, Losoff B, Maness J. Receptivity to library involvement in scientific data curation: a case study at the University of Colorado Boulder. portal: Libraries and the Academy. 2011; 11(4):915-937.
154. Laney CM, Pennington DD, Tweedie CE. Filling the gaps: sensor network use and data-sharing practices in ecological research. Frontiers in Ecology and the Environment. 2015; 13(7):363-368.
155. Lemke AA, Wolf WA, Hebert-Beirne J, Smith ME. Public and Biobank Participant Attitudes toward Genetic Research Participation and Data Sharing. Public Health Genomics. 2010; 13(6):368-77.
156. Lemke AA, Smith ME, Wolf WA, Trinidad SB. Broad data sharing in genetic research: views of institutional review board professionals. IRB: Ethics & Human Research. 2011; 33(3):1.
157. Longstaff H, Khramova V, Portales-Casamar E, Illes J. Sharing with more caring: coordinating and Improving the ethical governance of data and biomaterials obtained from children. PLoS One. 2015; 10(7):e0130527.
158. Lucas A, Palma-Dos-Reis A, Caldeira Mario. The quality of monitoring data in civil engineering works. 14th International Conference on Information Quality. 2009.
159. Luo A, Zheng K, Bhavnani S. Institutional infrastructure to support translational reserch. 2010 Sixth IEEE International Conference on e-Science. 2010. Available at: http://ieeexplore.ieee.org/document/5693898/?part=1. Accessed January 6, 2017.
160. Luzi D, Ruggieri R, Biagioni S, Schiano E. Data sharing in environmental sciences: A survey of CNR researchers. Fourteenth International Conference on Grey Literature National Research Council, Rome, Italy 29-30 November 2012. 2013. Available at: http://www.greynet.org/images/GL14-S2P,_Luzi_et_al.pdf. Accessed January 6, 2017.
161. Lyon L, Rusbridge C, Neilson C, Whyte A. Disciplinary Approaches to Sharing, Curation, Reuse and Preservation. 2010. Available at: http://www.dcc.ac.uk/sites/default/files/documents/scarp/SCARP-FinalReport-Final-SENT.pdf. Accessed January 6, 2017.
162. Magee AF, May MR, Moore BR. The dawn of open access to phylogenetic data. PLoS One. 2014; 9(10):e110268.
163. Manhas KP, Page S, Dodd SX Letourneau N, Ambrose A, Cui X, Tough SC. Parental perspectives on consent for participation in large-scale, non-biological data repositories. Life Sciences, Society and Policy. 2016; 12:1.
164. Manhas KP, Page S, Dodd SX, Letourneau N, Ambrose A, Cui X, Tough SC. Parent perspectives on privacy and governance for a pediatric repository of non-biological, research data. Journal of Empirical Research on Human Research Ethics. 2015; 10(1):88-99.
165. Manion FJ, Robbins RJ, Weems WA, Crowley RS. Security and privacy requirements for a multi-institutional cancer research data grid: an interview-based study. BMC Medical Informatics and Decision Making. 2009; 9:31.
166. Marcial LH, Hemminger BM. Scientific data repositories on the web: an initial survey. Journal of the American Society for Information Science and Technology. 2010; 61(10):2029-20292048.
167. Marcus C, Ball S, Delserone L, Hribar A, Loftus W. Understanding Research Behaviors, Information Resources, and Service Needs of Scientists and Graduate Students: A Study by the University of Minnesota Libraries. 2007. Available at: https://conservancy.umn.edu/handle/11299/5546. Accessed January 6, 2017.
168. Marshall B, O'Bryan K, Qin N, Vernon R. Organizing, Contextualizing, and Storing Legacy Research Data: A Case Study of Data Management for Librarians. Issues in Science and Technology Librarianship. 2013. Available at: http://www.istl.org/13-fall/article1.html. Accessed January 6, 2017.
169. Martinez-Uribe L. Digital Repository Services for Managing Research Data: What Do Oxford Researchers Need? IASSIST Quarterly. 2007; Fall/Winter:28-33.
170. Massey KA, Magee LA, Dale S, Claydon J, Morris TJ, von Dadelszen P, Liston RM, Ansermino JM. A current landscape of provincial perinatal data collection in Canada. Journal of Obstetrics and Gynaecology Canada. 2009; 31(3):236-246.
171. Mattern E, Jeng W, He D, Lyon L, Brenner A. Using participatory design and visual narrative inquiry to investigate researchers' data challenges and recommendations for library research data services. Program. 2015; 49(4):408-423.
172. Mayernik MS. Research data and metadata curation as institutional issues. Journal of the Association for Information Science and Technology. 2016; 67(4):973-993.
173. McDonald H, Nugent CD, Hallberg J, Finlay D, Moore, G. An approach for the creation of accessible and shared datasets. International Conference on Ubiquitous Computing and Ambient Intelligence. 2012; 224-232.
174. McGuire AL, Achenbaum LS, Whitney SN, Slashinski MJ, Versalovic J, Keitel WA, McCurdy SA. Perspectives on human microbiome research ethics. Journal of Empirical Research on Human Research Ethics. 2012; 7(3):1-14.
175. McGuire AL, Hamilton JA, Lunstroth R, McCullough LB, Goldman A. DNA data sharing: research participants' perspectives. Genetics In Medicine. 2008; 10(1):46-53.
176. McGuire AL, Oliver JM, Slashinski MJ, Graves JL, Wang T, Kelly PA, Fisher W, Lau CC, Goss J, Okcu M, Treadwell-Deering D, Goldman AM, Noebels JL, Hilsenbeck SG. To share or not to share: a randomized trial of consent for data sharing in genome research. Genetics In Medicine. 2011; 13(11):948-955.
177. McKay D. Oranges Are Not the Only Fruit: An Institutional Case Study Demonstrating Why Data Digital Libraries Are Not the Whole Answer to E-Research. International Conference on Asian Digital Libraries. 2010; 236-49.
178. McLure M, Level AV, Cranston CL, Oehlerts B, Culbertson M. Data curation: a study of researcher practices and needs. portal: Libraries and the Academy. 2014; 14(2):139-164.
179. Mello MM, Clarridge BR, Studdert DM. Academic medical centers' standards for clinical-trial agreements with industry. New England Journal of Medicine. 2005; 352:2202-10.
180. Menzies K, Birrell D, Dunsire G. New evidence on the interoperability of information systems within UK universities. International Conference on Theory and Practice of Digital Libraries. 2010; 104-115.
181. Milia N, Congiu A, Anagnostou P, Montinaro F, Capocasa M, Sanna E, Destro Bisol G. Mine, Yours, Ours? Sharing Data on Human Genetic Variation. PLoS One. 2012; 7(6):e37552.
182. Mills JA, Teplitsky C, Arroyo B, Charmantier A, Becker PH, Birkhead TR, Bize P, Blumstein DT, Bonenfant C, Boutin S, Bushuev A, Cam E, Cockburn A, Côté SD, Coulson JC, Daunt F, Dingemanse NJ, Doligez B, Drummond H, Espie RH, Festa-Bianchet M, Frentiu F, Fitzpatrick JW, Furness RW, Garant D, Gauthier G, Grant PR, Griesser M, Gustafsson L, Hansson B, Harris MP, Jiguet F, Kjellander P, Korpimäki E, Krebs CJ, Lens L, Linnell JD, Low M, McAdam A, Margalida A, Merilä J, Møller AP, Nakagawa S, Nilsson JÅ, Nisbet IC, van Noordwijk AJ, Oro D, Pärt T, Pelletier F, Potti J, Pujol B, Réale D, Rockwell RF, Ropert-Coudert Y, Roulin A, Sedinger JS, Swenson JE, Thébaud C, Visser ME, Wanless S, Westneat DF, Wilson AJ, Zedrosser A. Archiving primary data: solutions for long-term studies. Trends in Ecology & Evolution. 2015; 30(10):581-9.
183. Milner J. A UK research data service (UKRDS): the way forward for research data management? Serials. 2009; 22(1):83-5.
184. Minifie FD, Robey RR, Horner J, Ingham JC, Lansing C, McCartney JH, Alldredge EE, Slater SC, Moss SE. Responsible conduct of research in communication sciences and disorders: faculty and student perceptions. Journal of Speech, Language & Hearing Research. 2011; 54(1):S363-93.
185. MischoWH, Schlembach MC, O'Donnell MN. An Analysis of Data Management Plans in University of Illinois National Science Foundation Grant Proposals. Journal of eScience Librarianship. 2014; 3(1):31-43.
186. Mohr AH, Bishoff J, Bishoff C, Braun S, Storina C, Johston LR. When Data Is a Dirty Word: A Survey to Understand Data Management Needs Across Diverse Research Disciplines. Bulletin of the Association for Science and Technology. 2015; 42(1):51-53.
187. Mooney H, Newton MP. The Anatomy of a Data Citation: Discovery, Reuse,and Credit. Journal of Librarianship and Scholarly Communication. 2012; 1(1):eP1035.
188. Murillo AP. Data at risk initiative: examining and facilitating the scientific process in relation to endangered data. Data Science Journal. 2014; 12:207-219.
189. Murphy SN, Dubey A, Embi PJ, Harris PA, Richter BG, Turisco F, Weber GM, Tcheng JE, Keogh D. Current state of information technologies for the clinical research enterprise across academic medical centers. Clinical and Translational Science. 2012; 5(3):281-4.
190. Nicholson SW, Bennett TB. Data sharing: academic libraries and the scholarly enterprise. Portal: Libraries and the Academy. 2011; 11(1):505-516.
191. Noor MA, Zimmerman KJ, Teeter KC. Data Sharing: How Much Doesn’t Get Submitted to GenBank? PLoS Biology. 2006; 4(7):e228.
192. Noorman M, Kalaitzi V, Angelaki M, Tsoukala V, Linde P, Sveinsdottir T, Price L, Wessels B. Institutional evaluation and support for open access data policy. 2014. Available at: http://recodeproject.eu. Accessed January 6, 2017.
193. Oleksik G, Milic-Frayling N, Jones R. Beyond data sharing: Artifact ecology of a collaborative nanophotonics research centre. Proceedings of the ACM 2012 conference on Computer Supported Cooperative Work. 2012; 1165-1174.
194. Oliver JM, Slashinski MJ, Wang T, Kelly PA, Hilsenbeck SG, McGuire AL. Balancing the risks and benefits of genomic data sharing: genome research participants' perspectives. Public Health Genomics. 2012; 15(2):106-14.
195. Oushy MH, Palacios R, Holden AEC, Ramirez AG, Gallion KJ, O'Connell MA. To share or not to share? A survey of biomedical researchers in the U.S. Southwest, an ethnically diverse region. PLoS One. 2015; 10(9):e0138239.
196. Parsons T, Grimshaw S, Williamson L. Research Data Management Survey. 2013. Available at: http://eprints.nottingham.ac.uk/1893. Accessed January 6, 2017.
197. Pearce N. A study of technology adoption by researchers: web and e-science infrastructures to enhance research. Information, Communication & Society. 2010; 13(8):1191-1206.
198. Pejova P, Vyitalova H. Survey of enhanced publications in the Czech Republic. International Conference on Grey Literature. 2013; 15:91.
199. Pepe A, Mayernik M, Borman CL, Van de Sompel H. From Artifacts to Aggregations: Modeling Scientific Life Cycles on the Semantic Web. Journal of the American Society for Information Science and Technology (JASIST). 2010; 61(3):567-582.
200. Pepe A, Goodman A, Muench A, Crosas M, Erdmann C. How do astronomers share data? Reliability and persistence of datasets linked in AAS publications and a qualitative study of data practices among US astronomers. PloS One. 2014; 9(8):e104798.
201. Peters C, Dryden AR. Assessing the academic library's role in campus-wide research data management: a first step at the University of Houston. Science & Technology Libraries. 2011; 30(4):387-403.
202. Pham-Kanter G, Zinner DE, Campbell EG. Codifying collegiality: recent developments in data sharing policy in the life sciences. PLoS One. 2014; 9(9):e108451.
203. Pinfield S, Cox AM, Smith J. Research data management and libraries: relationships, activities, drivers and influences. PLoS One. 2014; 9(12):e114734.
204. Piwowar HA, Chapman WW. Public sharing of research datasets: A pilot study of associations. Journal of Informetrics. 2010; 4(2):148-156.
205. Piwowar HA, Chapman WW. A Review of Journal Policies for Sharing Research Data. Proceedings ELPUB 2008 Conference on Electronic Publishing. 2008. Available at: http://elpub.scix.net/data/works/att/001_elpub2008.content.pdf. Accessed January 6, 2017.
206. Piwowar HA. Who shares? Who doesn’t? Bibliometric factors associated with open archiving of biomedical datasets. ASIST 2010. 2010; 47(1):1-2.
207. Piwowar HA, Day RS, Fridsma DB. Sharing detailed research data is associated with increased citation rate. PLoS One. 2007; 2(3):e308.
208. Piwowar HA, Vision TJ. Data reuse and the open data citation advantage. PeerJ. 2013; 1:e175.
209. Piwowar HA. Who shares? Who doesn't? Factors associated with openly archiving raw research data. PLoS One. 2011; 6(7):e18657.
210. Polydoratou P. Use and Linkage of Source and Output Repositories and the Expectations of the Chemistry Research Community About Their Use. International Conference on Asia-Pacific Digital Libraries 2006. 2006. Available at: http://link.springer.com/chapter/10.1007%2F11931584_46. Accessed January 6, 2017.
211. Pope LC, Keyse J, Riginos C, Liggins L, Carvalho SB. Not the time or the place: the missing spatio-temporal link in publicly available genetic data. Molecular Ecology. 2015; 24(15):3802-9.
212. Procter R, Voss A, Asgari-Targhi M. Fostering the human infrastructure of e-research. Information, Communication & Society. 2013; 16(10):1668-1691.
213. Prost H, Malleret C, Schopfel J. Hidden Treasures: Opening Data in PhD Dissertations in Social Sciences and Humanities. Journal of Librarianship and Scholarly Communication. 2015; 3(2):eP1230.
214. Pryor G. Project StORe: making the connections for research. OCLC Systems and Services. 2007; 23(1):70-78.
215. Qin J, D'Ignazio J. The Central Role of Metadata in a Science Data Literacy Course. Journal of Library Metadata. 2010; 10:188-204.
216. Raju R, Schoombee L. Research support through the lens of transformation in academic libraries with reference to the case of Stellenbosch University Libraries. South African Journal of Libraries and Information Science. 2013; 79(2):27-38.
217. Rans J. Planning for the future: developing and preserving information resources in the Arts and Humanities. 2013; DCC RDM Services case studies. Edinburgh: Digital Curation Centre. Available at: http://www.dcc.ac.uk/resources/developing-rdm-services/dmps-arts-and-humanities. Accessed January 6, 2017.
218. Rathi V, Dzara K, Gross CP, Hrynaszkiewicz I, Joffe S, Krumholz HM, Strait KM, Ross JS. Sharing of clinical trial data among trialists: a cross sectional survey. BMJ. 2012; 345:e7570.
219. Ray B, Jackson C, Ducat E, Ho A, Hamon S, Kreek MJ. Effect of ethnicity, gender and drug use history on achieving high rates of affirmative informed consent for genetics research: impact of sharing with a national repository. Journal of Medical Ethics. 2011; 37(6):374-379.
220. Read KB, Surkis A, Larson C, McCrillis A, Graff A, Nicholson J, Xu J. Starting the data conversation: informing data services at an academic health sciences library. Journal of the Medical Library Association. 2015; 103(3):131-5.
221. Reidpath DD, Allotey PA. Data sharing in medical research: an empirical investigation. Bioethics. 2001; 15(2):125-34.
222. Richardson J, Nolan-Brown T, Loria P, Bradbury S. Library research support in Queensland: a survey. Australian Academic & Research Libraries. 2012; 43(4):258-277.
223. Rimkus K, Padilla T. Popp T, Martin G. Digital Preservation File Format Policies of ARL Member Libraries: An Analysis. D-Lib Magazine. 2014; 20(3/4). Available at: http://www.dlib.org/dlib/march14/rimkus/03rimkus.html. Accessed January 6, 2017.
224. Roos A. Developing Research Data Management Training and Support at Helsinki University Library. LIBER Case Study. 2014. Available at: http://libereurope.eu/wp-content/uploads/2014/06/LIBER-Case-Study-Helsinki.pdf. Accessed January 6, 2017.
225. Rostami R, Nahm M, Pieper CF. What can we learn from a decade of database audits? The Duke Clinical Research Institute experience, 1997-2006. Clinical Trials. 2009; 6(2):141-50.
226. Rousidis D, Garoufallou E, Balatsoukas P, Sicilia MA. Data quality issues and content analysis for research data repositories: The case of Dryad. 18th International Conference on Electronic Publishing. 2014; 49-58.
227. Samuel SM, Grochowski PF, Lalwani LN, Carlson J. Analyzing data management plans: Where librarians can make a difference. 2015 ASEE Annual Conference & Exposition. 2015; 26:215.
228. Sands AE, Borgman CL, Traweek S, Wynholds LA. We’re Working On It: Transferring the Sloan Digital Sky Survey from Laboratory to Library. International Journal of Digital Curation. 2014; 9(2):98-110.
229. Savage CJ, Vickers AJ. Empirical study of data sharing by authors publishing in PLoS journals. PLoS One. 2009; 4(9):e7078.
230. Sayogo DS, Pardo TA. Exploring the determinants of scientific data sharing: Understanding the motivation to publish research data Government Information Quarterly. 2013; 30(S1):S9-S31.
231. Sayogo DS, Pardo TA. Exploring the determinants of publication of scientific data in open data initiative. Proceedings of the 5th International Conference on Theory and Practice of Electronic Governance. 2011; 97-106.
232. Scaramozzino JM, Ramirez ML, McGaughey KJ. A study of faculty data curation behaviors and attitudes at a teaching-centered university. College & Research Libraries. 2012; 73(4):349-365.
233. Schmidt B, Ludwig J. Piloting Research Data Support at the University of Goettingen. 2014. Available at: http://libereurope.eu/wp-content/uploads/2014/07/LIBER-Case-Study-Goettingen.pdf. Accessed January 6, 2017.
234. Schmidt B, Gemeinholzer B, Treloar A. Open Data in Global Environmental Research: The Belmont Forum's Open Data Survey. PLoS One. 2016; 11(1):e0146695.
235. Schumacher J, VanderCreek D. Intellectual Capital at Risk: Data Management Practicesand Data Loss by Faculty Members at Five American Universities. International Journal of Digital Curation. 2015; 10(2):96-109.
236. Schwartz A, Pappas C, Sandlow LJ. Data repositories for medical education research: issues and recommendations. Academic Medicine. 2010; 85(5):837-843.
237. Shen Y. Research Data Sharing and Reuse Practices of Academic Faculty Researchers: A Study of the Virgina Tech Data Landscape. International Journal of Digitial Curation. 2015; 10(s):157-175.
238. Si, L, Xing W, Zhuang X, Hua X, Zhou L. Investigation and analysis of research data services in university libraries. Electronic Library. 2015; 33(3):417-49.
239. Silva LV, Goncalves MA, Laender AHF. Evaluating a digital library self-archiving service: The BDBComp user case study. Information Processing and Management. 2006; 43(4):1103-1120.
240. Simons N, Visser K, Searle S. Growing Institutional Support for Data Citation. D-Lib Magazine. 2013. Available at: http://www.dlib.org/dlib/november13/simons/11simons.html. Accessed January 6, 2017.
241. Simukovic E. Developing Research Data Management Services at Humboldt University Berlin. LIBER Case Study. 2014. Available at: http://libreas.eu/ausgabe23/10delasalle. Accessed January 6, 2017.
242. Snajdr E. Data curation in avian ecology: a case study from both the scientist's and librarian's view. International Association of Scientific and Technological University Linraries, 31st Annual Conference. 2010; Paper 2.
243. Soehner C, Steeves C, Ward J. E-science and data support services: a survey of ARL members. International Association of Scientific and Technological University Libraries, 31st Annual Conference. 2010; Paper 1.
244. Stamatolos A, Neville T, Henry D. Analyzing the Data Management Environment in a Master's-level Institution. Journal of Academic Librarianship. 2016; 42(2):154-160.
245. Stanton JM, Kim, Y, Oakleaf M, Lankes RD, Gandel P, Cogburn D, Liddy ED. Education for eScience professionals: job analysis, curriculum guidance, and program considerations. Journal of Education for Library and Information Science. 2011; 52(2):79-94.
246. Steinhart G, Chen E, Arguillas F, Kramer S. Prepared to Plan? A snapshot of researcher readiness to address data management planning requirements. Joural of eScience Librarianship. 2012; 1(2):63-78.
247. Strasser CA, Hampton SE. The fractured lab notebook: undergraduates and ecological data management training in the United States. Ecosphere. 2012; 3(12):1-18.
248. Sturges P, Bamkin M, Anders JHS, Hubbard B, Hussain A, Heeley M. Research Data Sharing: Developing a Stakeholder-Driven Model for Journal Policies. Journal of the Association for Information Science and Technology. 2014; 66(12):2445-2455.
249. Sulakhe D, Kettimuthu R, Dave U. High-performance data management for genome sequencing centers using globus online: a case study. E-Science (e-Science), 2012 IEEE 8th International Conference. 2012; 1-6.
250. Kim S, Lee W. Global data repository status and analysis: based on Korea, China and Japan Library Hi Tech. 2014; 32(4):706-722.
251. Tenopir C, Allard S, Douglass K, Aydinoglu AU, Wu L, Read E, Manoff M, Frame M. Data sharing by scientists: practices and perceptions. PLoS One. 2011; 6(6):e21101.
252. Tenopir C, Dalton ED, Allard S, Frame M, Pjesivac I, Birch B, Pollock D, Dorsett K. Changes in data sharing and data reuse practices and perceptions among scientists worldwide. PLoS One. 2015; 10(8):e0134826.
253. Tenopir C, Sandusky RJ, Allard S, Birch B. Academic librarians and research data services: preparation and attitudes. IFLA Journal. 2013; 70-78.
254. Trimble L, Woods C, Berish F, Jakubek D, Simpkin S. Collaborative Approaches to the Management of Geospatial Data Collections in Canadian Academic Libraries: A Historical Case Study. Western Libraries Publications. 2015; 11(3):330-358.
255. Tuyl S, Michalek G. Assessing Research Data Management Practices of Faculty at Carnegie Mellon University. Journal of Librarianship and Scholarly Communication. 2015; 3(3):eP1258.
256. Valentino M, Boock M. Data Management for Graduate Students: A Case Study at Oregon State University. Practical Academic Librarianship. 2015; 5(2):77-91.
257. Van den Eynden V, Bishop L. Sowing the seed: Incentives and motivations for sharing research data, a researcher’s perspective. 2014. Available at: http://www.data-archive.ac.uk/media/492924/ke_report-incentives-for-sharing-research-data.pdf. Accessed January 6, 2017.
258. Van Tuyl S, Whitmire AL. Water, water, everywhere: defining and assessing data sharing in academia. PLoS One. 2016; 11(2):e0147942.
259. van der Graaf M. The European Repository Landscape 2008. Inventory of digital repositories for research output. 2009.
260. Varvel Jr VE, Bammerlin EJ, Palmer CL. Education for data professionals: A study of current courses and programs. Proceedings of the 2012 iConference. 2012; 527-529.
261. Varvel VE Jr, Shen Y. Data management consulting at the Johns Hopkins University. New Review of Academic Librarianship. 2013; 19(3):224-245.
262. Verbaan E, Cox AM. Occupational sub-cultures, jurisdictional struggle and third space: theorising professional service responses to research data management. Journal of Academic Librarianship. 2014; 40(3-4):211-19.
263. Verbakel E. Essentials 4 Data Support. LIBER Case Study. 2014. Available at: http://libereurope.eu/wp-content/uploads/2014/06/LIBER-Case-Study-Essential-4-Data-Support.pdf. Accessed January 6, 2017.
264. Vines TH, Albert AYK; Andrew RL, Debarre F, Bock DG, Franklin MT, Gilbert KJ, Moore JS, Renaut S, Rennison DJ. The availability of research data declines rapidly with article age. Current Biology. 2014; 24(1):94-7.
265. Vines TH, Andrew RL, Bock DG, Franklin MT, Gilbert KJ, Kane NC, Moore JS, Moyers BT, Renaut S, Rennison DJ, Veen T, Yeaman S. Mandated data archiving greatly improves access to research data. FASEB Journal. 2013; 27(4):1304-18.
266. Vlaeminck S, Wagner GG. On the role of research data centres in the management of publication-related research data. Results of a survey among scientific infrastructure service providers in the field of social sciences. LIBER Quarterly. 2014; 23(4):336-57.
267. Voell MR, Farris L, Levy E, Marden E. A response to Rome: lessons from pre- and post-publication data-sharing in the C. elegans research community. BMC Genomics. 2010; 11:708.
268. Vogeli C, Yucel R, Bendavid E, Jones LM, Anderson MS, Louis KS, Campbell EG. Data Withholding and the Next Generation of Scientists: Results of a National Survey. Academic Medicine. 2006; 81(2):128-36.
269. Vrana R. Digital repositories of scientific information at the Croatian universities: Developing the bridge towards e-science. Proceedings of the ITI 2013 35th International Conference. 2013; 145-50.
270. Waddington S, Zhang J, Knight G, Jensen J, Downing R, Ketley C. Cloud repositories for research data-addressing the needs of researchers. Journal of Cloud Computing. 2013; 2:13.
271. Wallis JC, Borgman CL, Mayernik MS, Pepe A. Moving Archival Practices Upstream: An Exploration of the Life Cycle of Ecological Sensing Data in Collaborative Field Research. International Journal of Digitial Curation. 2008; 3(1):114-126.
272. Wallis JC, Rolando E, Borgman CL. If we share data, will anyone use them? Data sharing and reuse in the long tail of science and technology. PLoS One. 2013; 8(7):e67332.
273. Wang M, Fong BL. Embedded data librarianship: a case study of providing data management support for a science department. Science & Technology Libraries. 2015; 34(3):228-240.
274. Wang Y, Pakhomov S, Dale JL, Chen ES, Melton GB. Application of HL7/LOINC document ontology to a university-affiliated integrated health system research clinical data repository. AMIA Joint Summits on Translational Science Proceedings. 2014; 2014:230-234.
275. Weller T, Monroe-Gulick A. Understanding methodological and disciplinary differences in the data practices of academic researchers. Library Hi Tech. 2010; 32(3):467-482.
276. Weller T, Monroe-Gulick A. Differences in the Data Practices, Challenges, and Future Needs of Graduate Students and Faculty Members. Journal of eScience Librarianship. 2015; 4(1):e1070.
277. White HC. Considering Personal Organization: Metadata Practices of Scientists. Journal of Library Metadata. 2010; 10(2-3):156-172.
278. Whitmire AL, Boock M, Sutton SC. Variability in academic research data management practices. Program. 2015; 49(4):382-407.
279. Whyte A. Improving Research Visibility- Getting Data on the Institutional Repository RADAR. 2013; DCC RDM Services case studies. Edinburgh: Digital Curation Centre. Available at: http://www.dcc.ac.uk/resources/developing-rdm-services/repository-radar. Accessed January 6, 2017.
280. Wicherts JM, Bakker M, Molenaar D. Willingness to Share Research Data Is Related to the Strength of the Evidence and the Quality of Reporting of Statistical Result. PLoS One. 2011; 2011;6(11):e26828.
281. Wiley CA. An Analysis of Datasets within Illinois Digital Environment for Access to Learning and Scholarship (IDEALS), the University of Illinois Urbana-Champaign Repository. Journal of eScience Librarianship. 2015; 4(2):e1081.
282. Wilkinson M. Research Data Services at University College London. LIBER Case Study. 2014. Available at: http://libereurope.eu/wp-content/uploads/2014/06/LIBER-Case-Study-UCL.pdf. Accessed January 6, 2017.
283. Williams SC. Data practices in the crop sciences: a review of selected faculty publications. Journal of Agricultural & Food Information. 2012; 13(4):308-325.
284. Williams SC. Using a bibliographic study to identify faculty candidates for data services. Science & Technology Libraries. 2013; 32(2):202-209.
285. Willis C, Greenberg J, White H. Analysis and Synthesis of Metadata Goals for Scientific Data. Journal of the American Society For Information Science and Technology. 2012; 63(8):1505-1520.
286. Willoughby C, Bird CL, Coles SJ, Frey JG. Creating context for the experiment record. User-defined metadata: investigations into metadata usage in the LabTrove ELN. Journal of Chemical Information and Modeling. 2014; 54(12):3268-3283.
287. Wilson JA. University of Oxford Research Data Management Infrastructure.
      LIBER Case Study. 2014. Available at: http://libereurope.eu/wp-content/uploads/2014/06/LIBER-Case-Study-UOX.pdf. Accessed January 6, 2017.
288. Winget MA. Ramirez M. Developing a Meaningful Digital Self-Archiving Model: Archival Theory vs. Natural Behavior in the Minds of Carolina Research Project. Proceedings of the American Society for Information Science and Technology. 2004; 43(1):1-12.
289. Wright SJ, Kozlowski WA, Dietrich D, Khan HJ, Steinhart GS, McIntosh L. Using data curation profiles to design the datastar dataset registry. D-Lib Magazine. 2013; 19(7-8):37-49.
290. Wynholds L, Fearon Jr DS, Borgman CL, Traweek S. When use cases are not useful: Data practices, astronomy, and digital libraries. Proceedings of the 11th Annual International ACM/IEEE Joint Conference on Digital Libraries. 2011; 383-386.
291. Wynholds LA, Wallis JC, Borgman CL, Sands A, Traweek S. Data, data use, and scientific inquiry: Two case studies of data practices. Proceedings of the 12th ACM/IEEE-CS Joint Conference on Digital Libraries. 2012; 19-22.
292. Xia J. Mandates and the Contributions of Open Genomic Data. Publications. 2013; 1(3):99-112.
293. Xia J, Wang M. Competencies and responsibilities of social science data librarians: an analysis of job descriptions. College & Research Libraries. 2014; 75(3):362-388.
294. Yardley SJ, Watts KM, Pearson J, Richardson JC. Ethical issues in the reuse of qualitative data: perspectives from literature, practice, and participants. Qualitative Health Research. 2014; 24(1):102-113.
295. Yoon A. End users’ trust in data repositories: definition and influences on trust development. Archival Science. 2014; 14(1):17-35.
296. Kim Y, Zhang P. Understanding data sharing behaviors of STEM researchers: The roles of attitudes, norms, and data repositories. Library & Informaiton Science Research. 2015; 37(3):189-200.
297. Zachary I, Boren SA, Simoes E, Jackson-Thompson J, Davis JW, Hicks L. Information management in cancer registries: evaluating the needs for cancer data collection and cancer research. Online Journal of Public Health Informatics. 2015; 7(2):e213.
298. Zenk-Moltgen W, Leptein G. Data sharing in sociology journals. Online Information Review. 2014; 38(6):709-722.
299. Zhang T, Maron DJ, Charles CC. Usability evaluation of a research repository and collaboration web site. Journal of Web Librarianship. 2013; 7(1):58-82.
300. Zimmerman AS. Data Sharing and Secondary Use of Scientific Data: Experiences of Ecologists to locate data for reuse. PhD Dissertation. 2003.
301. Zinner DE, Pham-Kanter G, Campbell EG. The changing nature of scientific sharing and withholding in academic life sciences research: trends from national surveys in 2000 and 2013. Academic Medicine. 2016; 91(3):433-440.

**COMPANION ARTICLES**

1. Qin J, Solinger C. Institutional policies on science research data: a pilot analysis. Proceedings of the 2011 iConference. 2011;761-762.
2. Borgman CL, Darch PT, Sands AE, Wallis JC, Traweek S. The ups and downs of knowledge infrastructures in science: Implications for data management. Digital Libraries (JCDL), 2014 IEEE/ACM Joint Conference. 2014; 257-266.
3. Chao TC. Methods metadata: curating scientific research data for reuse. Doctoral dissertation. University of Illinois at Urbana-Champaign, 2015.
4. Burstein MD, Robinson JO, Hilsenbeck SG, McGuire AL, Lau CC. Pediatric data sharing in genomic research: attitudes and preferences of partents. Pediatrics. 2014;133(4):690-697.
5. Pryor G. Beyond publication - a passage through Project StOREe. Proceedings ELPUB2007 Conference on Electronic Publishing. 2007;107-116.
6. Rathi VK, Strait KM, Gross CP, Hrynaszkiewicz I, Joffe S, Krumbholz HM, Dzara K, Ross J. Predictors of clinical trial data sharing: exploratory analysis of a cross-sectional survey. Trials. 2014;15:384.
7. Shen Y. Strategic planning or a data-driven shared-access research enterprise: Virginia Tech research data assessment and landscape study. College and Research Libraries. 2016;77(4):500-519.
8. Soehner C, Steeves C, Ward J. e-Science and data support services: a study of ARL member institutions. August 2010. Available at: http://www.arl.org/storage/documents/publications/escience-report-2010.pdf. Accessed January 5, 2017.
9. Williams SC. Data sharing interviews with crop sciences faculty: why they share data and how the library can help. Issues in Science and Technology Librarianship. 2013. Available at: Available from: http://www.istl.org/13-spring/refereed2.html. Accessed January 4, 2017.
10. Zimmerman AS. Not by metadata along: the use of diverse forms of knowledge to locate data for reuse. Int J Digit Libr. 2007;7:5-16.
